# PSYCHOMETRIC PROPERTIES OF THE OLDER ADULT TECHNOPHOBIA SCALE AND SMARTPHONE CHALLENGE TASK

**DOI:** 10.1093/geroni/igz038.1195

**Published:** 2019-11-08

**Authors:** Kelly J Martin, Sean A Lauderdale, Steven Thorpe, Jan Molhman

**Affiliations:** 1 Texas A&M University-Commerce, Commerce, Texas, United States; 2 California School of Professional Psychology (CSPP) Alliant, San Diego, California, United States; 3 William Paterson University, Wayne, New Jersey, United States

## Abstract

Older adults are especially prone to anxiety if they are unable to keep pace with technological advances and are generally more technophobic than their younger counterparts. Older adults tend to limit their use of technology, if not avoid it altogether, such as using a smartphone for calls and text messages only, while eschewing more advanced functions. Currently, there is no measure of technophobia in older adults that captures fears and concerns about the use of these up-to-date technological tools. The purpose of this investigation was to evaluate the psychometric properties of a new scale of technophobia and corresponding smartphone challenge task in a sample of older adults. Community-dwelling older adults (N = 42, 81.0% female, Mage = 77.3) completed the following: the Older Adult Smartphone Challenge Task (OASCT), Older Adults’ Technophobia Scale (OATS), Older Adult Social Anxiety Scale, Computer Anxiety Rating Scale, and the IPIP Five Factor Personality Domains. Preliminary data indicate good internal consistency for the OATS (α = .87) and the OASCT (α = .86). The OASCT was negatively correlated with age, computer anxiety, and OATS anxiety/avoidance scores, but positively correlated with education. The OATS scores were positively correlated with social anxiety, social avoidance, and computer anxiety, but negatively correlated with extraversion. To keep pace with the contemporary world, older adults must achieve a level of comfort with the use of technological devices. Administering the OASCT and OATS could be a valuable first step in identifying older adults with technology-related deficits and anxiety for individual and/or community-wide intervention.

